# Multilayer Graphene Stacked with Silver Nanowire Networks for Transparent Conductor

**DOI:** 10.3390/ma18010208

**Published:** 2025-01-06

**Authors:** Jinsung Kwak

**Affiliations:** 1 Department of Physics, Changwon National University, Changwon 51140, Republic of Korea; jkwak@changwon.ac.kr; 2 Department of Materials Convergence and System Engineering, Changwon National University, Changwon 51140, Republic of Korea

**Keywords:** multilayer graphene, silver nanowire, flexible transparent electrode, chemical vapor deposition

## Abstract

A mechanically robust flexible transparent conductor with high thermal and chemical stability was fabricated from welded silver nanowire networks (w-Ag-NWs) sandwiched between multilayer graphene (MLG) and polyimide (PI) films. By modifying the gas flow dynamics and surface chemistry of the Cu surface during graphene growth, a highly crystalline and uniform MLG film was obtained on the Cu foil, which was then directly coated on the Ag-NW networks to serve as a barrier material. It was found that the highly crystalline layers in the MLG film compensate for structural defects, thus forming a perfect barrier film to shield Ag NWs from oxidation and sulfurization. MLG/w-Ag-NW composites were then embedded into the surface of a transparent and colorless PI thin film by spin-coating. This allowed the MLG/w-Ag-NW/PI composite to retain its original structural integrity due to the intrinsic physical and chemical properties of PI, which also served effectively as a binder. In view of its unique sandwich structure and the chemical welding of the Ag NWs, the flexible substrate-cum-electrode had an average sheet resistance of ≈34 Ω/sq and a transmittance of ≈91% in the visible range, and also showed excellent stability against high-temperature annealing and sulfurization.

## 1. Introduction

Current research on optoelectronic devices is pursuing low-cost, multifunctional, highly efficient devices with good long-term stability. Transparent electrodes (TEs) are one of the essential components in optoelectronic device applications, including solar cells, organic light-emitting diodes, touch screen panels, and liquid crystal displays [1,2,3]. In the past, indium tin oxide (ITO) was used in almost every electronic device as a TE due to its excellent optoelectrical properties [1,2]. However, despite its high performance, there are still many disadvantages in using ITO to produce low-cost devices. For example, high raw material costs and expensive deposition methods drive up total manufacturing costs; for instance, indium is a rare metal with limited sources. Depositing the ITO film onto a substrate is also an expensive and time-consuming process. Commercial ITO film is fabricated by a direct current sputtering process in a vacuum system, rather than using a cheaper deposition method such as a solution process. In addition, TEs fabricated using ITO show weak mechanical properties, especially under bending loads [4].

To overcome the limitations of ITO, various alternative nanomaterials including carbon nanotubes (CNTs), graphene (Gr), conducting polymers (CPs), metal nanowires (NWs), and metal grids have been explored [5,6,7,8,9,10]. Among these various alternatives, one-dimensional (1D) metal NWs are promising candidates to replace ITO, since TEs with 1D metal NWs can exhibit excellent optoelectronic and electronic properties [1,2,11]. However, there are certain drawbacks that need to be resolved before these materials can be used in practical applications. Taking the synthesis of silver (Ag) NWs as an example, the high contact resistance caused by insulating polymer residues and the high surface roughness which results from the stacking of Ag NWs into networks can cause performance degradation and electrical shorts in optoelectronic devices, respectively [12,13,14]. And metal NW films often suffer from instability due to aging, oxidation, and sulfurization when exposed to various environmental conditions [15]. Such degradation of Ag NW electrodes at the wire junctions can cause sharp increases in electrical resistance due to the formation of AgO, Ag_2_O, or Ag_2_S [16]. Large efforts have been dedicated to overcoming these problems. For example, low- and high-temperature welding, chemical welding, or plasmonic welding processes can reduce the junction resistance, while combining Ag NWs with other conducting materials such as Gr, graphene oxide (GO), or CPs can induce a smoother surface [17,18,19,20,21,22]. Nevertheless, it has been difficult to completely overcome the intrinsic limitations of metal NWs for long-term usage. One promising approach to overcome this problem would be to introduce a highly crystalline two-dimensional (2D) barrier material which can enhance the long-term stability and optoelectrical properties of a metal NW electrode.

Graphene, a highly crystalline 2D carbon (C) material, is known to have excellent optoelectrical and mechanical properties and to be inherently impermeable to almost all atoms and molecules due to its highly crystalline hexagonal structure [23,24,25]. However, its ideal properties are only evident in its pristine form obtained by mechanical exfoliation methods from graphite crystals [26,27,28,29]; furthermore, the process of exfoliation is not amenable for scalable production. Currently, the chemical vapor deposition (CVD) method can offer significantly improved, highly crystalline graphene structures on a catalytic metal substrate. Various TE applications have been reported using CVD-grown large-area graphene [1,2]; however, despite significant efforts from numerous research groups, low sheet resistance cannot be reproducibly obtained using graphene alone at this moment. Instead, there have been attempts to introduce graphene as a barrier film to protect metal films from oxidation. There have been reports of introducing reduced GO (rGO), which is easy to produce in large quantity, applied together with Ag NWs to reduce the surface roughness and enhance the environmental stability [30]. However, inherent defects in rGO make it ineffective at enhancing the long-term environmental stability and reducing the surface roughness without sacrificing the transmittance of the composite film. In contrast, highly crystalline CVD-grown single-layer graphene (SLG) on a Cu foil can provide better transmittance with a low level of defects. Still, atomic-scale defects and the troublesome wet-transfer process can result in tears and wrinkles which cannot completely protect the Ag NW networks from degradation [31,32].

Therefore, it is necessary to develop a method of depositing highly crystalline multilayer graphene (MLG) for protecting Ag NWs from degradation. Significant efforts have been made to develop an MLG growth process using a CVD method on various metal substrates [33]. Generally, a metal catalyst with low carbon solubility (e.g., Cu) is an excellent candidate for growing uniform, large-area, highly crystalline SLG due to the surface-mediated graphene growth mechanism [34,35]. In contrast, metal catalysts with high C solubility (e.g., Ni, Pt, Pd, Fe, etc.) induce non-uniformities in MLG due to its bulk diffusion growth mechanism [36,37,38,39,40]. Several groups have reported MLG growth using Ni or Cu-Ni alloys [38,39,40]; however, these methods require precise control over the catalyst composition and have complicated growth parameters to control, such as methane (CH_4_) feed rate and cooling rate. Recently, MLG growth on Cu was reported by modifying the Cu foil into a Cu envelope to obtain highly crystalline and uniform MLG [41,42]. With such a method, the growth rate of graphene can be effectively modified on the inner and outer surfaces of the Cu envelope by altering the growth atmosphere. This asymmetric graphene growth on the opposite sides of a Cu surface can result in a different C concentration gradient through the bulk Cu matrix, which can induce the diffusion of C from the inner to the outer surface. Although highly crystalline and uniform MLG was obtained using a Cu envelope with a CVD method, it is yet not applicable for practical applications due to its long synthesis time (>12 h), and the need for a Cu envelop limits the scalability of this method of producing MLG.

In this work, we developed a sandwich structured MLG/Ag-NW/polyimide (PI) composite film for high-performance and environmentally stable electrodes for TE applications. First, MLG was grown using a low-pressure CVD (LPCVD) method on a Cu foil. The MLG growth was obtained by simply loading Cu foil on the bottom of a quartz tube leaving a 1 cm gap. Modifying the gas flow dynamics and surface chemistry of the Cu substrate (i.e., changing the gap distance between the Cu foil and quartz tube and oxidizing the Cu surface before CVD growth, respectively) induced different graphene growth rates on each side of the Cu, and asymmetric graphene growth was achieved within a relatively short growth time. As a result, highly crystalline MLG with an average coverage of ≈97% was grown on the top surface of the Cu foil and then applied to Ag NWs as an anti-oxidation barrier film. Second, we developed a direct transfer method to apply MLG/Ag-NWs onto a transparent PI film by casting a soluble PI solution onto Ag-NW/MLG/Cu foil structures followed by an etching process to remove the metal catalyst. With a unique sandwich structure and a chemical welding process for the Ag NWs, the composite film presented an average sheet resistance (*R*_sh_) of ≈34 Ω/sq and a transmittance of ≈91% in the visible range and showed excellent stability against oxidation and sulfurization. The resulting MLG/Ag-NW/PI composite films were stable under ambient conditions at least for 90 days, which makes them applicable for TE applications requiring long-term stability.

## 2. Materials and Methods

### 2.1. Growth of MLG on a Cu Foil

The CVD method was used to grow MLG on a 25 μm thick Cu foil (98% purity, Alfa Aesar, Ward Hill, MA, USA). In brief, a Cu foil was electropolished in an aqueous phosphoric acid solution for 15 min to produce a smooth surface and to remove surface contamination. After electropolishing, the Cu foil was rinsed with distilled (DI) water followed by an isopropyl alcohol (IPA) treatment. Before the CVD growth process, the electropolished Cu foil (i.e., as-prepared Cu) was annealed in air at 200 °C for 30 min by using a hot plate (i.e., air-oxidized Cu). The air-oxidized Cu foil was placed at the bottom of the quartz tube in our CVD system. The temperature was raised to ~1050 °C within 35 min with 200 sccm H_2_ gas flow. This was maintained for 1 h. Prior to the growth step, a diluted O_2_ (1000 ppm in Ar) gas flow of 200 sccm was introduced for 10 min (i.e., CVD-oxidized Cu). In addition to the oxidation of the Cu surface, these processes help to flatten the Cu surface, but also remove various carbon contaminants from the Cu surface through forming gaseous CO*_x_* [39]. Finally, MLG layers were grown by introducing 1 sccm CH_4_ and 200 sccm H_2_ gas flow for 2 h, respectively.

### 2.2. Synthesis of Ag NWs Using a Continuous Flow Method

Using a homemade glass tubular reactor, Ag-NWs were synthesized through a continuous flow method. Silver nitrate (AgNO_3_, ≥99.0%), sodium chloride (NaCl, ≥99.0%), sodium bromide (NaBr, ≥99.0%), polyvinylpyrrolidone (PVP, molecular weight: 360,000), and ethylene glycol (EG, anhydrous, 99.8%) were purchased from Sigma Aldrich (St. Louis, MO, USA) and used without further purification. In brief, we synthesized Ag NWs through a modified polyol fabrication method. The first step was to dissolve 1.008 g of PVP in 30 mL of EG. Next, two sets of seeding solutions of NaCl and NaBr were prepared separately at 0.5 mM molar concentrations. After PVP was fully dissolved in an EG solvent, appropriate amounts of the seeding solutions were added and mixed well for 1 h at room temperature. Then, 0.509 g of AgNO_3_ was dissolved in an EG solution containing PVP, NaCl, and NaBr, for 10 min at room temperature. For the fabrication of Ag-NWs, the resulting mixture was transferred to a glass syringe and the solution was introduced continuously into a reactor by using a syringe pump. In this process, the temperature of the continuous flow reactor was set at ≈160 °C. By adjusting the injection speed of the syringe pump, the retention time was precisely controlled.

### 2.3. Fabrication of MLG/Ag-NW/PI TEs

The MLG/Ag-NW/PI composite was fabricated by first spin-coating the Ag-NW solution (0.3 mg/mL) onto an MLG/Cu foil after the CVD process. Next, using a solution of AgNO_3_ dissolved in distilled water (0.035 mM), chemical welding of Ag-NWs was carried out. This aqueous solution was first dropped onto the Ag-NW/MLG/Cu foil with a residual time of 15 s and spin-coated for 60 s at 2000 rpm. After welding the Ag-NWs, a PI solution dissolved in dimethylformamide (DMF, 30 wt%) was spin-coated onto a welded Ag-NW/MLG/Cu foil (i.e., w-Ag-NW/MLG/Cu) for direct formation of the TE composite. To evaporate the solvent and ensure good adhesion between the w-Ag-NW/MLG/Cu foil and PI film, the PI-coated composite was sequentially heated at 50 °C for 30 min, 100 °C for 1 h, and 150 °C for 1 h by using a hot plate. Finally, using an aqueous ammonium persulfate solution, the Cu foil was etched away.

### 2.4. Characterization of MLG/w-Ag-NW/PI Composite

A field emission scanning electron microscope (FE-SEM, Hitachi-S-4800, Hitachi, Tokyo, Japan) was used to observe the surface morphologies of the as-synthesized MLG, Ag-NWs, and MLG/(w-)Ag-NW/PI composites. To measure the electromechanical stability of the MLG/PI, (w-)Ag-NW/PI, and MLG/(w-)Ag-NW/PI composites, each composite was prepared with a size of ≈2 × 2 cm^2^ and was clamped on the homemade bending tester. Whilst repeatedly bending the films, we observed a change in the resistance of various TE composites, in which the radius of curvature was set to 3 mm. The structural characteristics of the as-synthesized MLG layers and the stacking order were investigated by Raman spectroscopy. The Raman spectra were measured on a WiTec alpha 300R M-Raman system with an excitation wavelength of 532 nm; the laser power was ~1 mW and the laser spot size was ~640 nm using a ×50 objective lens with a numerical aperture of 0.5. The transmittances of the composite TEs were measured by using UV-Vis-NIR spectroscopy at a wavelength range of 350~800 nm (Cary 5000 model, Varian, Palo Alto, CA, USA). The sheet resistances (*R*_sh_) were measured by using a Hall-effect system with the four-point probe method (EM4-HVA model).

## 3. Results

### 3.1. Fabrication and Structural Characterization of MLG/Ag-NW/PI Sandwich Structure

**Figure 1:**
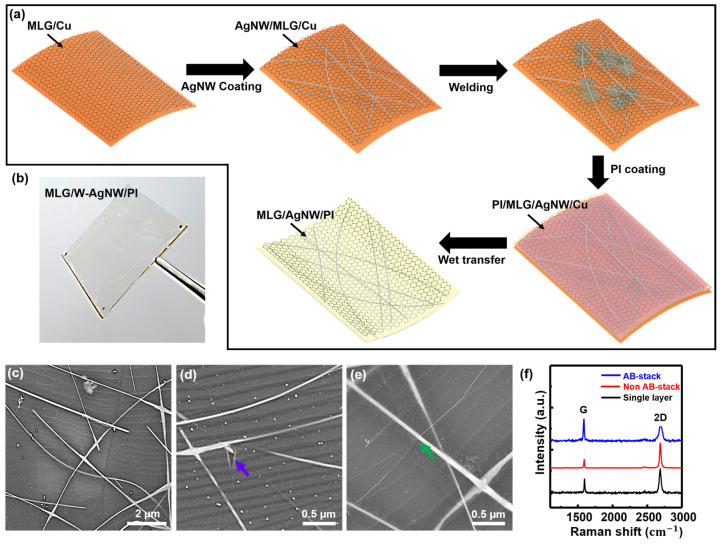
(**a**) A sequential schematic diagram of fabricating MLG/Ag-NW/PI composites. First, using LPCVD in our oxidation protocols, a large-area MLG layer was grown on a Cu foil. Then, Ag-NW solution was spin-coated on an MLG/Cu foil. Followed by chemical welding of Ag-NW networks on an MLG/Cu foil, transparent soluble PI solution was spin-coated and cured for structural integrity. Finally, Cu was etched away and the MLG/w-Ag-NW/PI composite was obtained. (**b**) A photo of MLG/w-Ag-NW/PI held by tweezers. (**c**–**e**) Representative SEM images of MLG/w-Ag-NW/PI composite. Purple arrow in (**d**) indicates typical torn MLG and green arrow in (**e**) indicates a wrinkle of MLG right above Ag-NWs. (**f**) Representative Raman spectrum obtained from various locations in an MLG layer.

We have developed a method to directly coat Ag NW networks onto graphene without forming any unintentional defects, such as tearing and folding graphene, which occur under the conventional wet-transfer process of graphene [31]. A schematic illustration of the MLG/w-Ag-NW/PI composite fabrication process is shown in Figure 1a. First, MLG was grown on a Cu foil using the LPCVD method, and details of this will be shown in Section 3.2. The Ag NWs were then spin-coated onto an MLG/Cu sample. The density of the Ag NWs was controlled by changing the number of spin-coating treatments. Next, a chemical welding process was applied to ensure tight contact between each Ag NW for low junction resistance. Subsequently, a solution of PI in DMF solvent was spin-coated over the w-Ag-NW/MLG/Cu composite. A final annealing step was applied to prepare an integrated structure. It was noticed that, in this annealing process, slow elevation of the temperature was important to ensure the integrity and uniformity of the composite film, and PI was chosen as the substrate for its high transparency and hydrophobic surface that adheres well on graphene layers. Finally, the MLG/w-Ag-NW/PI composite with high performance was obtained after etching the Cu away using an APS etchant solution (Figure 1b). The MLG/w-Ag-NW/PI composite was observed using SEM. The images confirm that MLG was incorporated with the Ag NW networks into a sandwich structure (Figure 1c). The Ag NW networks were buried well under MLG, as confirmed by the observation of tears (Figure 1d) and wrinkles of MLG crossing over the Ag NWs (Figure 1e). It was noted that Ag NWs were either interconnected to each other by direct crossing or in contact with MLG. Some of the Ag NWs were buried under transparent PI, which is shown in the SEM images with pale gray contrast. Such a unique structure is beneficial for the protection of Ag NW networks from degradation under various environmental conditions. The MLG was analyzed using Raman spectroscopy. In Figure 1f, highly crystalline MLG is confirmed from negligible D peaks around 1350 cm^−1^. By analyzing 100 randomly chosen spots, it was found that ~68% of the MLG was AB-stacked (in which the 2D peak around 2700 cm^−1^ has an asymmetric shape, a blue line), whereas ~32% of the MLG was randomly oriented (in which the 2D peak around 2700 cm^−1^ has a symmetric shape, a red line).

### 3.2. Growth of Graphene with High Multilayer Coverage on a Cu Foil

Several recent reports have indicated that the metal catalytic substrate plays an important role in graphene growth kinetics [33,34,35,36,37,38,39,40,41,42]. The mechanism of MLG growth is the subject of much debate, especially when Cu substrates are involved [43,44,45,46,47]. In general, MLG can be grown either (i) by diffusion from underneath (i.e., back surface of a Cu foil) to the top graphene layer (i.e., front surface of a Cu foil) [41,42], or (ii) by a gas-phase penetration of C molecules through the graphene edges on the front surface of a Cu foil [43]. When a Cu envelope is used as a substrate for graphene growth, some insist that O molecules participate in MLG growth by adsorbing onto the Cu surface, forming CuO, Cu(OH)_2_, and Cu_2_O. These species affect not only the graphene growth rate, resulting in asymmetric graphene growth, but also lower the CH_4_ dissociation energy barrier and activate C diffusion from the opposite side of the Cu surface, resulting in MLG flakes [44,45]. Conversely, there have been reports on the interaction between the graphene edges and Cu surfaces when the hydrogen partial pressure is adjusted. A high hydrogen partial pressure can induce bonding between graphene edges and hydrogen atoms, causing it to detach from the Cu surface. Consequently, C atoms penetrate the interface between the graphene and Cu substrate and become MLG [46]. Likewise, O can have a similar effect on MLG growth. The Cu_2_O layer formed during graphene growth can decouple the graphene from the Cu surface and facilitate the penetration of C molecules [47].

**Figure 2:**
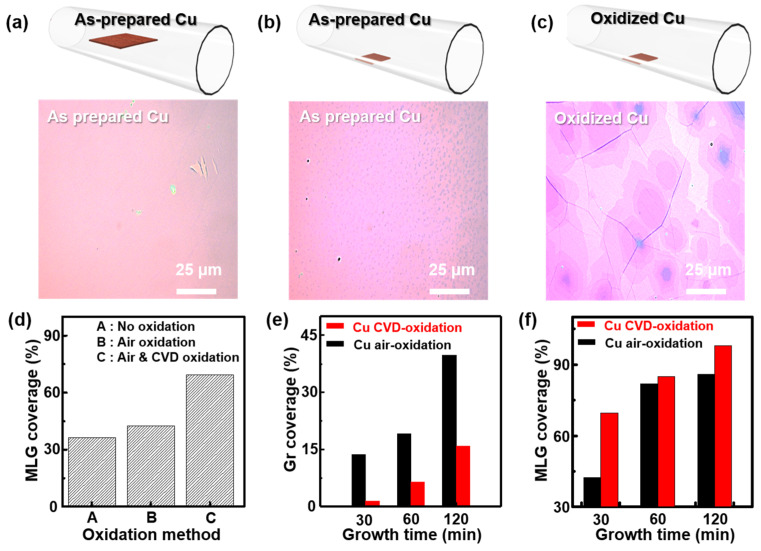
(**a**,**b**) Schematic illustrations of layer-controlled CVD graphene synthesis by changing the position of a Cu foil loaded in the interior of a quartz tube (upper panels) and corresponding optical microscope images of as-synthesized graphene layers transferred onto a Si/SiO_2_ substrate (lower panels). (**c**) A schematic illustration of an air-oxidized Cu foil loaded at the bottom of a quartz tube with MLG transferred onto a Si/SiO_2_ substrate observed using an optical microscope. (**d**) MLG coverage data as a function of Cu foil oxidation method. (**e**) Data on graphene coverage on back side of Cu foil as a function of Cu foil oxidation method. (**f**) MLG coverage data as a function of growth time.

Although the exact mechanism of MLG growth is unclear at this moment, our MLG growth strategy was aimed at inducing asymmetric graphene growth on the front and back surfaces of a Cu foil with the incorporation of O molecules, which can facilitate complete dehydrogenation of CH*_x_* and MLG growth by diffusion of C atoms through the Cu. According to the Hagen–Poiseuille law, the flow rate of a fluid is quadratically proportional to the cross-sectional area [48]. Several graphene samples were grown on each Cu foil, which was located at different heights in a CVD quartz tube. As can be seen from Figure 2a,b, the as-prepared Cu foil was put either at the center or at the bottom of the quartz tube to examine whether the location of the Cu foil can induce asymmetric graphene growth on its front and back surfaces. When the Cu foil is loaded at the bottom of the quartz tube, we suggest that there is a significant difference in the volumetric gas flow rate between the front and back sides of the Cu foil, according to the Hagen–Poiseuille law. For this reason, above the Cu foil, the boundary layer thickness for mass transfer is much thinner than that beneath the Cu foil. Therefore, the significant difference in the mass flux impinged on the front and back surfaces of the Cu foil provokes the gas precursor concentration gradient during graphene growth, resulting in an asymmetric growth rate of graphene in the two regions. Therefore, after graphene growth, the different loading locations showed different graphene growth behaviors. For instance, when the Cu foil was loaded at the center of the quartz tube, full coverage of SLG was observed on the front and back sides of the Cu foil (Figure 2a). However, when the Cu foil was put at the bottom of the quartz tube, full coverage of SLG with MLG flakes was observed on the front side of the Cu foil, while only partial growth of graphene was observed on the back side (Figure 2b). We believe that asymmetric graphene growth on the Cu foil loaded at the bottom of the quartz tube allowed C diffusion from the back side of the Cu foil and resulted in MLG flakes. In contrast, symmetric graphene growth was observed on the Cu foil loaded at the center of the quartz tube; the Cu foil was completely covered with SLG, which inhibited further diffusion of C atoms from the back side of the Cu foil. To enlarge the domain size of MLG flakes, we introduced air-oxidized Cu and loaded it at the bottom of the quartz tube for LPCVD graphene growth. We noticed that enlarged MLG domains with flake sizes of ≈20 µm were successfully grown on the Cu foil (Figure 2c). As discussed earlier, O incorporation can help MLG growth; however, it is not clear whether this MLG growth was due to the effect of O gas or the Cu foil location, the latter of which induces asymmetric graphene growth.

As shown in Figure 2d, it can be noted that oxidized Cu tends to show a higher coverage of MLG on the front surface of the Cu foil. This result is correlated with back-side graphene coverage, where oxidized Cu tends to present a lower coverage of graphene on the back side of the Cu foil (Figure 2e). Since heavily oxidized Cu tends to show lower graphene coverage on the back side when the Cu foil is loaded at the bottom of a quartz tube, it can be assumed that a lower coverage of graphene on the back side of a Cu foil can induce more C atoms to diffuse and result in larger MLG. With such conditions, we were able to enhance MLG coverage on the front side of a Cu foil by placing oxidized Cu at the bottom of the quartz tube and increasing the growth time. When the growth time was prolonged to 2 h, almost full coverage of MLG (≈97%) was obtained on the front side of the Cu foil (Figure 2f).

### 3.3. Electrical and Mechanical Properties of MLG/w-Ag NW/PI Composite

**Figure 3:**
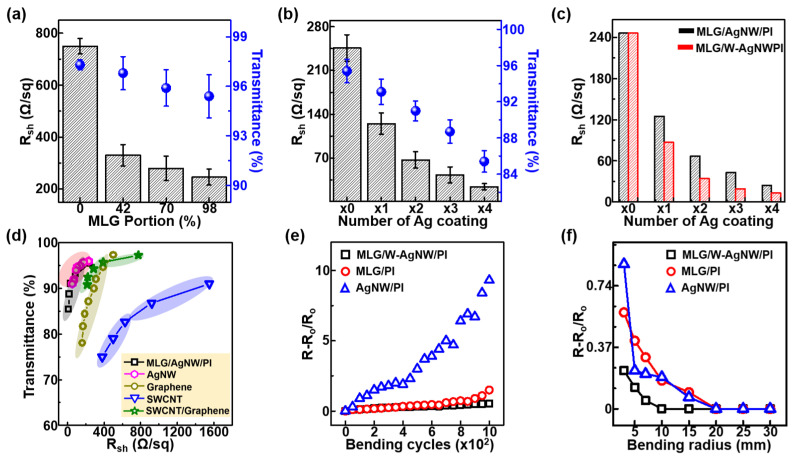
(**a**) Sheet resistance and transmittance at 550 nm of Gr/PI composites according to the MLG portion. (**b**) Sheet resistance and transmittance at 550 nm of MLG/Ag-NW/PI films depending on the number of Ag coating cycles, in which MLG coverage is ~98%. (**c**) Comparison of sheet resistance values before and after chemical welding of Ag-NWs on the Ag-NW/MLG/Cu foil. (**d**) Comparison of optoelectronic properties (*R*_sh_ and transmittance at 550 nm) between our MLG/w-AgNW/PI composite and previously reported results. The performance of Ag-NW [40], graphene [41], SWCNT [42], and SWCNT/graphene [43] composites is shown for comparison. (**e**,**f**) Mechanical bending properties of TCEs upon continuous bending cycles and bending radii, respectively.

Figure 3a illustrates the sheet resistance (*R*_sh_) values of CVD-grown SLG and MLG measured using the van der Pauw method with a Hall measurement apparatus. Monolayer-dominant graphene exhibited a value of ≈750 ± 30 Ω/sq with ≈97.3 ± 0.3% transmittance in the visible range. As the MLG portion on SLG increased from ≈42 to ≈70 and ≈98%, the *R*_sh_ and transmittance in the visible range also decreased from ≈330 ± 41 to ≈280 ± 47 and ≈246 ± 31 Ω/sq and ≈96.8 ± 1.0, ≈95.9 ± 1.1, and ≈95.4 ± 1.3%, respectively. Since a high coverage of MLG is necessary to protect Ag-NW networks, an Ag-NW solution was spin-coated onto an MLG/Cu foil one to four times, in which MLG coverage was around 97%. As shown in Figure 3b, both *R*_sh_ and transmission in the visible range are gradually decreased as the number of coatings is increased and, after coating the Ag-NW solution four times, changed to ≈24 ± 5 Ω/sq with ≈85.4 ± 1.2%. It has been reported that the optoelectronic properties of Ag NWs largely depend on contact between each NW [17,18,19,20,21,22,49]. To enhance the electrical contact between Ag NWs, a simple chemical welding process was applied by treating the Ag NWs with a AgNO_3_ solution. The AgNO_3_ solution treatment simultaneously provides Ag^0^ at the junctions of Ag NWs through galvanic displacement and causes the fusing of these junctions. Figure 3c compares the *R*_sh_ values of the MLG/Ag -NW/PI composites before and after the chemical welding process. When the welding process was applied one to four times on Ag NWs coated with MLG, reductions in *R*_sh_ of ≈69.6, ≈50.7, ≈44.1, and ≈42% were observed; most importantly, *R*_sh_ could be reduced from ≈67 to ≈34 Ω/sq, while maintaining a transmittance of ≈91 ± 1.1%. As shown in Figure 3d, our MLG/w-Ag-NW/PI composite showed outstanding performance compared to other TEs based on Ag NWs, graphene, SWCNT or SWCNT/graphene composites [49,50,51,52].

The sandwich structure of the MLG/w-Ag-NW/PI composite also has the benefit of mechanical flexibility because the continuous PI and MLG strongly hold the Ag-NW networks while under strain. Using a homemade bending tester, we tested the mechanical stability of MLG, Ag-NWs, and MLG/w-Ag-NW/PI composites under bending. As can be seen from Figure 3e, throughout 1000 bending cycles at a bending radius of ≈3 mm, the ∆R of the MLG/w-Ag-NW/PI composite remained stable, where ∆*R* = (*R*_f_ − *R*_i_)/*R*_i_ (*R*_f_: final resistance value; *R*_i_: initial resistance value). Unlike Ag-NW/PI and MLG/PI composites, the ∆R of the MLG/w-Ag-NW/PI composite started to increase when the bending radius was ≈10 mm and induced a 23% change in ∆*R* at a maximum bending radius of ≈3 mm (Figure 3f). Generally, the increased resistance of graphene or Ag-NW-based TEs under mechanical strain is interpreted as the generation of line defects or detachment of the conducting materials from the substrate. We believe that the partially embedded structure of Ag NWs was held in place by PI and MLG, which acted as a binder, allowing the composite to retain its original structural integrity under bending stress.

### 3.4. Chemical Stability Under Various Enviromental Conditions

**Figure 4:**
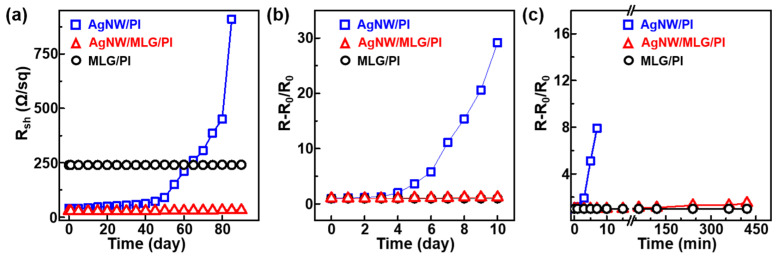
Test for environmental stability of our MLG/w-Ag-NW/PI composite in air (**a**) at ~25 °C and (**b**) ~100 °C and (**c**) in an aqueous ammonium persulfate solution (0.1 M), in which a change in resistance is observed.

To assess their chemical stability, TEs (MLG/PI, Ag-NW/PI, and MLG/w-Ag-NW/PI) were exposed to ambient, high temperature, or APS solution conditions, and ∆R was analyzed. The first step was to leave the composite films at room temperature for 90 days. As shown in Figure 4a, the MLG/PI and MLG/w-Ag-NW/PI films were very stable and did not exhibit any noticeable changes in *R*_sh_ after exposure to ambient conditions for 90 days; however, as time passed, the Ag-NW/PI composite film showed a gradual increase in *R*_sh_. Similarly, only the Ag-NW/PI composite film showed a significant increase in resistance when the TEs were tested at 100 °C for 10 days. On the other hand, the MLG/w-Ag-NW/PI and MLG/PI composites exhibited negligible changes in ∆R (Figure 4b). Finally, sulfurization tests were performed for each TE. When the Ag-NW/PI composite was treated with an APS solution, an immediate sharp increase in ∆R was observed, and the device shorted at 7 min (Figure 4c). However, the MLG/PI and MLG/w-Ag-NW/PI composites stayed stable for up to 7 h of treatment. The sulfurization test was extended to 24 h for the MLG/w-Ag-NW/PI composite and it showed only small changes in ∆R up to a maximum of 20%. These results suggest that highly crystalline MLG can be used as an effective barrier film for Ag NW networks in ambient conditions below ~100 °C. Additionally, unavoidable structural defects included in each graphene layer are probably canceled out by the highly crystalline MLG structures and act as an enhanced barrier film to shield Ag-NWs from oxidation and sulfurization.

## 4. Conclusions

TEs with high performance and long-term stability were formed using the MLG/Ag-NW/PI composite fabricated by a one-step process. By engineering the CVD growth parameters, we grew highly crystalline MLG with an average coverage of ≈97% on a Cu foil, which acted as an excellent barrier film for protecting Ag-NW networks. The high mechanical flexibility of the MLG/Ag-NW/PI composite was attributed to the partially embedded structure of Ag-NWs under the transparent PI and MLG, which enhanced the structural integrity of the composite. In addition, the highly crystalline and uniform MLG layer was determined to be an effective barrier film for Ag-NW networks under various environmental conditions. We believe that the performance of our MLG-based TEs can be further improved by obtaining uniform thickness of the as-synthesized graphene layers through the optimization of the graphene growth conditions.

## Data Availability

The data presented in this study are available on request from the corresponding author due to privacy.

## References

[1] Ellmer K. (2012). Past achievements and future challenges in the development of optically transparent electrodes. Nat. Photonics.

[2] Morales-Masis M., De Wolf S., Woods-Robinson R., Ager J.W., Ballif C. (2017). Transparent electrodes for efficient optoelectronics. Adv. Electron. Mater..

[3] Althumayri M., Das R., Banavath R., Beker L., Achim A.M., Koydemir H.C. (2024). Recent advances in transparent electrodes and their multimodal sensing applications. Adv. Sci..

[4] Oh S.J., Kwon J.H., Lee S., Choi K.C., Kim T.-S. (2021). Unveiling the annealing-dependent mechanical properties of freestanding indium tin oxide thin films. ACS Appl. Mater. Interfaces.

[5] Gruner G. (2006). Carbon nanotube films for transparent and plastic electronics. J. Mater. Chem..

[6] Kim C.-L., Jung C.-W., Oh Y.-J., Kim D.-E. (2017). A highly flexible transparent conductive electrode based on nanomaterials. NPG Asia Mater..

[7] Xu Y., Liu J. (2016). Graphene as transparent electrodes: Fabrication and new emerging applications. Small.

[8] Kim Y.H., Sachse C., Machala M.L., May C., Muller-Meskamp L., Leo K. (2011). Highly conductive PEDOT:PSS electrode with optimized solvent and thermal post-treatment for ITO-free organic solar cells. Adv. Funct. Mater..

[9] Lee J., Lee P., Lee H.B., Hong S., Lee I., Yeo J., Lee S.S., Kim T.S., Lee D., Ko S.H. (2013). Room-temperature nanosoldering of a very long metal nanowire network by conducting-polymer-assisted joining for a flexible touch-panel application. Adv. Funct. Mater..

[10] Kim C., An K., Kang M., Won P., Park J.-J., Cho K.H., Ko S.H., Ju B.-K., Kang K.-T. (2022). Facile fabrication of flexible metal grid transparent electrode using inkjet-printed dot array as sacrificial layer. Sci. Rep..

[11] Sciacca B., Groep J.V.D., Polman A., Garnett E.C. (2016). Solution-grown silver nanowire ordered arrays as transparent electrodes. Adv. Mater..

[12] Lee J., Lee I., Kim T.-S., Lee J.-Y. (2013). Efficient welding of silver nanowire networks without post-processing. Small.

[13] Jeong J.-M., Sohn M., Bang J., Lee T.-I., Kim M.-S. (2023). Fast, facile and thermal damage free nanowelding of Ag nanowire for flexible transparent conductive film by pressure-assisted microwave irradiation. Sci. Rep..

[14] Nam S., Song M., Kim D.-H., Cho B., Lee H.M., Kwon J.-D., Park S.-G., Nam K.-S., Jeong Y., Kwon S.-H. (2014). Ultrasmooth, extremely deformable and shape recoverable Ag nanowire embedded transparent electrode. Sci. Rep..

[15] Choo D.C., Kim T.W. (2017). Degradation mechanisms of silver nanowire electrodes under ultraviolet irradiation and heat treatment. Sci. Rep..

[16] Mayousse C., Celle C., Fraczkiewicz A., Simonato J.-P. (2015). Stability of silver nanowire based electrodes under environmental and electrical stresses. Nanoscale.

[17] Ge Y., Duan X., Zhang M., Mei L., Hu J., Hu W., Duan X. (2018). Direct room temperature welding and chemical protection of silver nanowire thin films for high performance transparent conductors. J. Am. Chem. Soc..

[18] Lee H.-J., Oh S., Cho K.-Y., Jeong W.-L., Lee D.-S., Park S.-J. (2018). Spontaneous and selective nanowelding of silver nanowires by electrochemical ostwald ripening and high electrostatic potential at the junctions for high-performance stretchable transparent electrodes. ACS Appl. Mater. Interfaces.

[19] Garnett E.C., Cai W., Cha J.J., Mahmood F., Connor S.T., Christoforo M.G., Cui Y., McGehee M.D., Brongersma M.L. (2012). Self-limited plasmonic welding of silver nanowire junctions. Nat. Mater..

[20] Chen R., Das S.R., Jeong C., Khan M.R., Janes D.B., Alam M.A. (2013). Co-percolating graphene-wrapped silver nanowire network for high performance, highly stable, transparent conducting electrodes. Adv. Funct. Mater..

[21] Meenakshi P., Karthick R., Selvaraj M., Ramu S. (2014). Investigations on reduced graphene oxide film embedded with silver nanowire as a transparent conducting electrode. Sol. Energy Mater. Sol. Cells.

[22] Yun H.J., Kim S.J., Hwang J.H., Shim Y.S., Jung S.-G., Park Y.W., Ju B.-K. (2016). Silver nanowire-IZO-conducting polymer hybrids for flexible and transparent conductive electrodes for organic light-emitting diodes. Sci. Rep..

[23] Zhao Y., Lin L. (2024). Graphene, beyond lab benches. Science.

[24] Othman M.A.K., Guclu C., Capolino F. (2013). Graphene-based tunable hyperbolic metamaterials and enhanced near-field absorption. Opt. Express.

[25] Pianelli A., Kowerdziej R., Dudek M., Sielezin K., Olifierczuk M., Parka J. (2020). Graphene-based hyperbolic metamaterial as a switchable reflection modulator. Opt. Express.

[26] Ishikawa R., Findlay S.D., Seki T., Sánchez-Santolino G., Kohno Y., Ikuhara Y., Shibata N. (2018). Direct electric field imaging of graphene defects. Nat. Commun..

[27] Banhart F., Kotakoski J., Krasheninnikov A.V. (2011). Structural defects in graphene. ACS Nano.

[28] Zhang Y., Zhang H., Li F., Shu H., Chen Z., Sui Y., Zhang Y., Ge X., Yu G., Jin Z. (2016). Invisible growth of microstructural defects in graphene chemical vapor deposition on copper foil. Carbon.

[29] Kwak J., Jo Y., Park S.-D., Kim N.Y., Kim S.-Y., Shin H.-J., Lee Z., Kim S.Y., Kwon S.-Y. (2017). Oxidation behavior of graphene-coated copper at intrinsic defects of different origins. Nat. Commun..

[30] Yang Y., Chen S., Li W., Li P., Ma J., Li B., Zhao X., Ju Z., Chang H., Xiao L. (2020). Reduced Graphene Oxide Conformally Wrapped Silver Nanowire Networks for Flexible Transparent Heating and Electromagnetic Interference Shielding. ACS Nano.

[31] Kwak J., Kim S.-Y., Jo Y., Kim N.Y., Kim S.Y., Lee Z., Kwon S.-Y. (2018). Unraveling the water impermeability discrepancy in CVD-grown graphene. Adv. Mater..

[32] Ahn Y., Jeong Y., Lee Y. (2012). Improved thermal oxidation stability of solution-processable silver nanowire transparent electrode by reduced graphene oxide. ACS Appl. Mater. Interfaces.

[33] Xue X., Wang L., Yu G. (2021). Surface Engineering of Substrates for Chemical Vapor Deposition Growth of Graphene and Applications in Electronic and Spintronic Devices. Chem. Mater..

[34] Li X., Cai W., An J., Kim S., Nah J., Yang D., Piner R., Velamakanni A., Jung I., Tutuc E. (2009). Large-area synthesis of high-quality and uniform graphene films on copper foil. Science.

[35] Zhao P., Kumamoto A., Kim S., Chen X., Hou B., Chiashi S., Einarsson E., Ikuhara Y., Maruyama S. (2013). Self-limiting chemical vapor deposition growth of monolayer graphene from ethanol. J. Phys. Chem. C.

[36] Xue Y., Wu B., Guo Y., Huang L., Jiang L., Chen J., Geng D., Liu Y., Hu W., Yu G. (2011). Synthesis of large-area, few-layer graphene on iron foil by chemical vapor deposition. Nano Res..

[37] Choi J.-K., Kwak J., Park S.-D., Yun H.D., Kim S.-Y., Jung M., Kim S.Y., Park K., Kang S., Kim S.-D. (2015). Growth of wrinkle-free graphene on texture-controlled platinum films and thermal-assisted transfer of large-scale patterned graphene. ACS Nano.

[38] Garcia J.M., He R., Jiang M.P., Kim P., Pfeiffer L.N., Pinczuk A. (2011). Multilayer graphene grown by precipitation upon cooling of nickel on diamond. Carbon.

[39] Chen S., Cai W., Piner R.D., Suk J.W., Wu Y., Ren Y., Kang J., Ruoff R.S. (2011). Synthesis and characterization of large-area graphene and graphite films on commercial Cu–Ni alloy foils. Nano Lett..

[40] Liu Z.-D., Yin Z.-Y., Du Z.-H., Yang Y., Zhu M.-M., Xie L.-H., Huang W. (2014). Low temperature growth of graphene on Cu-Ni alloy nanofibers for stable, flexible electrodes. Nanoscale.

[41] Fang W., Hsu A.L., Caudillo R., Song Y., Birdwell A.G., Zakar E., Kalbac M., Dubey M., Palacios T., Dresselhaus M.S. (2013). Rapid identification of stacking orientation in isotopically labeled chemical-vapor grown bilayer graphene by raman spectroscopy. Nano Lett..

[42] Fang W., Hsu A.L., Song Y., Birdwell A.G., Amani M., Dubey M., Dresselhaus M.S., Palacios T., Kong J. (2014). Asymmetric growth of bilayer graphene on copper enclosures using low-pressure chemical vapor deposition. ACS Nano.

[43] Gan L., Zhang H., Wu R., Zhang Q., Ou X., Ding Y., Sheng P., Luo Z. (2015). Grain size control in the fabrication of large single-crystal bilayer graphene structures. Nanoscale.

[44] Hao Y., Bharathi M.S., Wang L., Liu Y., Chen H., Nie S., Wang X., Chou H., Tan C., Fallahazad B. (2013). The role of surface oxygen in the growth of large single-crystal graphene on copper. Science.

[45] Hao Y., Wang L., Liu Y., Chen H., Wang X., Tan C., Nie S., Suk J.W., Jiang T., Liang T. (2016). Oxygen-activated growth and bandgap tunability of large single-crystal bilayer graphene. Nat. Nanotechnol..

[46] Zhang X., Wang L., Xin J., Yakobson B.I., Ding F. (2014). Role of hydrogen in graphene chemical vapor deposition growth on a copper surface. J. Am. Chem. Soc..

[47] Li J., Wang D., Wan L.-J. (2015). Unexpected functions of oxygen in a chemical vapor deposition atmosphere to regulate graphene growth modes. Chem. Commun..

[48] Chang R.-J., Lee C.-H., Lee M.-K., Chen C.-W., Wen C.-Y. (2017). Effects of surface oxidation of Cu substrates on the growth kinetics of graphene by chemical vapor deposition. Nanoscale.

[49] Yun H.D., Kwak J., Kim S.-Y., Seo H., Bang I.C., Kim S.Y., Kang S., Kwon S.-Y. (2016). High performance all-carbon composite transparent electrodes containing uniform carbon nanotube networks. J. Alloys Compd..

[50] Li B., Ye S., Stewart I.E., Alvarez S., Wiley B.J. (2015). Synthesis and purification of silver nanowires to make conducting films with a transmittance of 99%. Nano Lett..

[51] Wang Y., Tong S.W., Xu X.F., Ozyilmaz B., Loh K.P. (2011). Interface engineering of layer-by-layer stacked graphene anodes for high-performance organic solar cells. Adv. Mater..

[52] Shin D.-W., Lee J.H., Kim Y.-H., Yu S.M., Park S.-Y., Yoo J.-B. (2009). A role of HNO_3_ on transparent conducting film with single-walled carbon nanotubes. Nanotechnology.

